# Comparison of Early and Long-Term Mortality in Patients With Reduced and Preserved Ejection Fraction Undergoing Coronary Artery Bypass Graft: A Systematic Review and Meta-Analysis

**DOI:** 10.7759/cureus.43245

**Published:** 2023-08-09

**Authors:** Jithin Karedath, Sumreen Nazly, Syeda Fatima Murtaza, Sagar Bhandari, Anmol Sharma, Saifullah Talpur, Muhammad Moiz Vistro, Sujith K Palleti

**Affiliations:** 1 Internal Medicine, King's College Hospital NHS Foundation Trust, London, GBR; 2 Internal Medicine, University Medical and Dental College Faisalabad, Faisalabad, PAK; 3 Internal Medicine, Mayo Clinic, Jacksonville, USA; 4 Internal Medicine, Allama Iqbal Medical College, Lahore, PAK; 5 Cardiology, Tucson Medical Center, Tucson, USA; 6 Internal Medicine, B.P. Koirala Institute of Health Sciences, Dharan, NPL; 7 Psychiatry, Liaquat National Hospital and Medical College, Karachi, PAK; 8 Internal Medicine, United Medical and Dental College, Creek General Hospital, Karachi, PAK; 9 Nephrology, Edward Hines Jr. Veterans Administration Hospital, Hines, USA; 10 Nephrology, Loyola University Medical Center, Maywood, USA

**Keywords:** systematic review and meta-analysis, mortality, coronary artery bypass grafting(cabg), normal ejection fraction, reduced ejection fraction

## Abstract

The aim of this study was to compare early and long-term mortality in patients with reduced and preserved ejection fraction (EF) undergoing coronary artery bypass graft (CABG). This meta-analysis followed the Preferred Reporting Items for Systematic Reviews and Meta-Analyses (PRISMA) 2020 guidelines. Two investigators independently conducted a systematic and comprehensive search of PubMed, EMBASE, and Scopus from inception to July 15, 2023, using the search terms "reduced ejection fraction," "preserved ejection fraction," "coronary artery bypass surgery," and "mortality." Boolean operators (AND, OR) were used with medical subject heading (MeSH) terms to refine the search. The reference lists of all included articles were manually searched to identify potentially relevant studies. We restricted our search to studies published in the English language. The outcomes assessed in this meta-analysis included short-term mortality (including in-hospital and 30-day mortality) and long-term mortality. A total of five studies were included in this meta-analysis. The pooled sample size is 94,399 participants. Pooled analysis showed that the risk of early mortality was significantly higher in patients with reduced EF compared to patients with preserved EF (risk ratio, RR: 2.14, 95% CI: 1.50 to 3.06). The pooled analysis also reported that late mortality was significantly higher in patients with reduced EF compared to patients with preserved EF (RR: 1.67, 95% CI: 1.35 to 2.08). The pooled analysis of studies demonstrated a significantly higher rate of both early and late mortality in patients with reduced EF, emphasizing the importance of EF assessment in risk stratification for CABG patients.

## Introduction and background

For over four decades, coronary artery bypass graft (CABG) surgery has been a vital approach to alleviate symptoms and enhance survival in patients with coronary artery disease. Nonetheless, it is not without risks. Presently, the all-cause mortality rate stands at 1% for patients with preserved ejection fraction (EF), increasing to 7% for those with severely reduced EF [1]. Managing patients with low EF remains challenging, although CABG has shown superiority over medical therapy alone, leading to significant clinical improvement and long-term survival [2,3]. However, patients with reduced EF experience higher postoperative morbidity and mortality rates compared to those with normal left ventricular function [4]. Notably, advances in patient selection, surgical techniques, and pre-operative optimization have contributed to improved CABG outcomes [5]. Additionally, contemporary cardiac support devices like intra-aortic balloon pump (IABP), left ventricular assist device (LVAD), and Impella offer targeted therapy for acute cardiogenic shock, providing crucial hemodynamic support in critical situations. While these devices have distinct applications, such as LVADs for long-term heart failure management, their effectiveness in enhancing long-term survival remains a subject of ongoing research [5].

Coronary artery bypass grafting (CABG) has demonstrated greater efficacy in patients with compromised left ventricular function. Alderman et al. reported that compared to medical treatment alone, CABG significantly improved survival in patients with low EF [6,7]. Specifically, those with ejection fractions below 26% showed a substantial survival benefit after surgery, with a 63% five-year mortality rate compared to the 43% five-year mortality rate of medical therapy [6,8]. Moreover, patients with EF<50% who underwent CABG had a significantly higher seven-year survival rate (84%) than those on guideline-directed medical therapy (70%) [9]. Patients with impaired left ventricular function undergoing CABG form a distinct group, where risk factors for postoperative mortality may differ from those with normal EF. Christakis and colleagues [4] found that the urgency of surgery was the sole independent predictor of operative mortality in patients with an EF of <20% who underwent CABG.

Studies comparing survival in patients with reduced and preserved EF undergoing CABG are limited, and therefore, we are conducting this study to assess pooled estimates of survival in this group of patients. The aim of this meta-analysis is to compare early and long-term mortality in patients with reduced and preserved EF undergoing CABG.

## Review

Methodology

This meta-analysis followed the Preferred Reporting Items for Systematic Reviews and Meta-Analyses (PRISMA) 2020 guidelines.

Data Sources and Search Strategy

Two investigators independently conducted a systematic and comprehensive search of PubMed, EMBASE, and Scopus from inception to July 15, 2023, using the search terms "reduced ejection fraction," "preserved ejection fraction," "coronary artery bypass surgery," and "mortality." Boolean operators (AND, OR) were used with medical subject heading (MeSH) terms to refine the search. The reference lists of all included articles were manually searched to identify potentially relevant studies. We restricted our search to studies published in the English language. All records identified from the online database search were imported into EndNote X9 software (Clarivate Analytics, London, UK) and duplicates were removed. The titles and abstracts were screened, and full texts of eligible articles were obtained for detailed assessment based on pre-defined inclusion and exclusion criteria.

Eligibility Criteria

Articles were considered eligible for inclusion in this meta-analysis if they compared mortality in patients with reduced EF (EF<50%) and preserved EF (EF≥50%), with or without the presence of heart failure. We included randomized control trials (RCTs) and observational studies. We excluded studies that did not report the required outcomes. Additionally, case series, editorials, letters, protocols, reviews, and expert opinions were excluded from this systematic review. The outcomes assessed in this meta-analysis included short-term mortality (including in-hospital and 30-day mortality) and long-term mortality.

Data Extraction and Quality Assessment

The following data were extracted from the included studies: first name, year of publication, country of origin, study design, sample size, and participant characteristics, including age, gender, diabetes, hypertension, recurrent myocardial infarction, and dyslipidemia. One author extracted the data using the pre-designed data extraction form in a Microsoft Excel Spreadsheet (The Microsoft Corporation, Washington, USA), and a second author cross-checked it and entered it into Review Manager Software (The Cochrane Collaboration, London, UK) for analysis. The quality assessment of the included studies was performed using the Newcastle-Ottawa Scale. Three domains were scored, including selection of study groups, comparability of study groups, and assessment of outcomes.

Data Synthesis and Statistical Analysis

To compare the risk of mortality between patients with reduced and preserved EF and undergoing CABG, we performed an analysis using a random effects model. This model was selected due to the heterogeneity among the included studies, which involved variations in the study population and outcomes. For mortality, we computed risk ratios (RR). To compare baseline characteristics between preserved and reduced EF patients, risk ratios (RR) were calculated for categorical variables, and the mean difference (MD) was calculated for continuous variables. For each pooled estimate, a 95% confidence interval (CI) was calculated to assess the precision of the results. Heterogeneity was assessed using the I-square and Q-statistics, with an I-square value of less than 25% representing low heterogeneity, 25 to 50% representing moderate heterogeneity, and more than 50% representing high heterogeneity [9]. The level of statistical significance was set at 0.05.

Results

**Figure 1:**
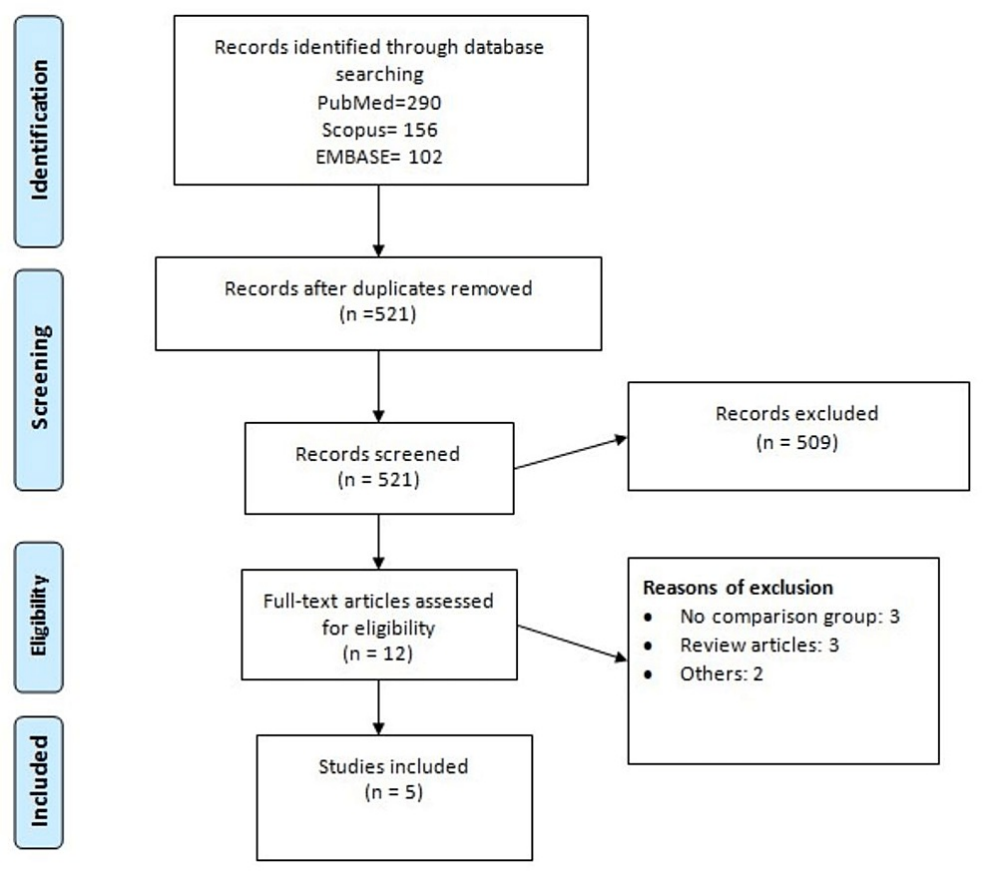
PRISMA flowchart of study selection. PRISMA: Preferred Reporting Items for Systematic Reviews and Meta-Analyses.

The database search yielded 548 records. After reviewing the titles and abstracts, 12 full texts were obtained. Out of 12 records, five studies fulfilled the inclusion and exclusion criteria and were included in the analysis [5,10-13]. Figure 1 shows the process of study selection. Table 1 shows the characteristics of the included studies. The pooled sample size is 94,399 participants. Two separate studies analyzed two cohorts of participants independently. As a result, in this meta-analysis, we analyzed these studies twice [11,12]. Table 2 presents the quality of the included studies.

**Table 1:** Characteristics of included studies.

Author Name	Year	Region	Groups	Total
Awan et al. [10]	2020	Pakistan	Reduced	589
			Preserved	625
Dalen et al. [11]	2016	Sweden	Reduced	3456
			Preserved	1216
Dalen et al. [11]	2017	Sweden	Reduced	10,069
			Preserved	27,165
Hamad et al. [5]	2010	Netherlands	Reduced	2081
			Preserved	8204
Sun et al. [12]	2018	Canada	Reduced	4816
			Preserved	2752
Sun et al. [12]	2019	Canada	Reduced	10,284
			Preserved	22,231
Thuijs et al. [13]	2020	United States	Reduced	115
			Preserved	796

**Table 2:** Quality assessment of included studies. +: Symbol represents one score.

Author name	Selection	Comparability	Outcomes	Overall
Awan et al. [10]	+++	+	+++	Good
Dalen et al. [11]	+++	++	+++	Good
Dalen et al. [11]	+++	++	+++	Good
Hamad et al. [5]	++	++	+++	Good
Sun et al. [12]	+++	++	+++	Good
Sun et al. [12]	+++	++	+++	Good
Thuijs et al. [13]	+++	++	+++	Good

Comparison of the Baseline Characteristics of Preserved Versus Reduced EF Patients

We compared the characteristics of patients with preserved and reduced EF patients. No significant difference was found between the two groups in terms of age (p-value: 0.70). Number of males was significantly greater in patients with reduced EF (p-value<0.0001). Additionally, comorbidities, including recurrent myocardial infarction and diabetes mellitus, were also significantly higher in patients with reduced EF compared to patients with preserved EF. However, being hypertensive is significantly associated with preserved ejection fraction, as the number of patients with hypertension is significantly greater in patients with preserved EF, as shown in Table 3.

**Table 3:** Comparison of baseline characteristics of participants. EF: ejection fraction; MI: myocardial infarction; RR: risk ratio; CI: confidence interval. ^: presented as mean. *: presented as mean difference (95% CI).

Variable	Reduced EF (%)	Preserved EF (%)	RR (95% CI)
Age^	65.75	65.54	0.21 (−0.83, 1.24)*
Male	81.28	77.81	1.08 (1.05, 1.11)
Dyslipidemia	28.13	31.94	0.87 (0.82, 0.92)
Hypertension	59.39	62.77	0.93 (0.89, 0.96)
Diabetes mellitus	40.53	31.77	1.04 (1.05, 1.23)
Prior MI	67.22	40.24	1.54 (1.34, 1.76)

Comparison of Early Mortality Between Preserved Versus Reduced EF Patients

**Figure 2:**
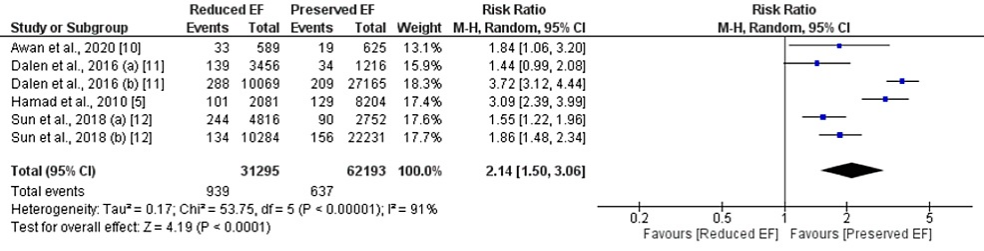
Early mortality. EF: Ejection fraction. Sources: References [5,9-12].

Four studies were included in the pooled analysis of comparing early mortality between patients with preserved EF and reduced EF encompassing 81836 patients (31295 with reduced EF and 62193 with preserved EF). As shown in Figure 2, the risk of early mortality was significantly higher in patients with reduced EF (n=939) compared to patients with preserved EF (n=637) (RR: 2.14, 95% CI: 1.50 to 3.06). Significant heterogeneity was reported among the study results.

Comparison of Long-term Mortality Between Preserved Versus Reduced EF Patients

**Figure 3:**
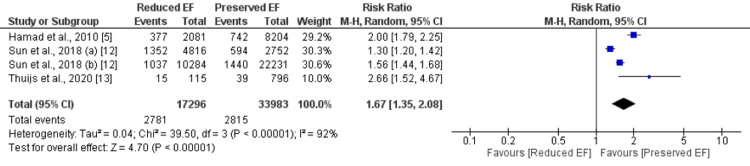
Long-term mortality. EF: Ejection fraction. Sources: References [5,12,13].

Three studies were included in the pooled analysis of comparing late mortality between patients with preserved EF and reduced EF. The pooled analysis reported that late mortality was 1.80 times significantly higher in patients with reduced EF compared to patients with preserved EF (16.07% vs. 8.28%) (RR: 1.67, 95% CI: 1.35 to 2.08), as shown in Figure 3. Significant heterogeneity was reported among the study results. 

Meta-regression

We performed the meta-regression to understand the factors causing heterogeneity in early mortality, and the results are shown in Table 4. The variable heart failure seems to be statistically significant, while age, diabetes, hypertension, dyslipidemia, and recurrent myocardial infarction do not appear to have a statistically significant impact on the outcome under study.

**Table 4:** Meta-regression. MI: myocardial infarction.

Variable	P-value
Age	0.655
Diabetes	0.334
Hypertension	0.252
Heart failure	0.035
Dyslipidemia	0.155
Recurrent MI	0.155

Discussion

This meta-analysis was conducted to compare mortality in patients with reduced and preserved EF undergoing CABG. The pooled analysis of five studies showed that the rate of early and late mortality is significantly higher in patients with reduced EF compared to patients with preserved EF. Reduced EF signifies a diminished ability of the left ventricle to eject blood with each contraction, resulting in decreased cardiac output and compromised overall cardiovascular function. This impaired heart function is associated with a decreased ability to deliver sufficient oxygen and nutrients to meet the body's demands, particularly during periods of stress or increased workload, such as during surgery [14]. Additionally, patients with reduced EF often have a higher burden of comorbidities, such as myocardial infarction and other cardiovascular conditions, which can further exacerbate the risk of adverse outcomes following CABG [15].

A low EF has been proven to be one of the risk factors for increased operative mortality [16]. In our study, we reported that the early mortality rate in patients with an EF of less than 50% was 2.14 times higher than in patients with an EF of more than 50%. This finding corroborates the outcomes of previous research on the initial impact of isolated CABG on mortality among patients with a low EF. For instance, the study conducted by Di Carli et al. [17] found a 9.3% 30-day mortality rate in patients with an EF of less than 20%, and the study conducted by Carr and colleagues [18] showed an 11% perioperative mortality rate in patients with an EF of 10 to 20%. Additionally, we found that heart failure is one of the risk factors that may influence our study results. The study conducted by Dalen et al. [11] and Sun et al. [12] reported poor survival in patients with heart failure undergoing CABG. However, due to the limited number of studies, validation of these findings is necessary to explore more factors associated with the outcome. 

Individuals with diminished left ventricular function who undergo CABG represent a unique patient cohort. The risk factors contributing to higher postoperative mortality rates in these patients may differ from those typically observed in individuals with normal EF levels. In their study, Christakis et al. [4] noted that among patients with an EF of less than 20% who underwent CABG, the urgency of the surgery was the sole independent predictor of operative mortality. On the other hand, different researchers [19] reported that for patients with an impaired EF who underwent CABG, an age of over 70 years was the only independent predictor of in-hospital mortality. In the current study, we also found that patients with a low EF had a higher incidence of comorbidities such as diabetes and myocardial infarction compared to patients with a normal EF. These factors may have contributed to the increased incidence of early mortality in patients with a low EF. The findings of our study are consistent with the results reported by Hamad et al. [5]. Previous studies also reported that hypertension, diabetes, obesity, and peri-operative outcomes are some of the predictors of poor outcomes among patients undergoing CABG [20-22]. On the other hand, the number of hypertensive patients was found to be higher in patients with preserved EF. A study conducted by Suzuki et al. reported that higher blood pressure was associated with EF improvement, even with optimal medical therapy [23].

The present study has certain limitations. Firstly, due to the lack of individual-level data, we were unable to perform subgroup analysis to determine how different covariates affect the outcome variable. Secondly, high heterogeneity was reported among the study results. To identify the reasons for heterogeneity, we performed meta-regression. Lastly, only one of the five included studies assessed medication records. Therefore, we were not able to assess the potential role of medications on the outcomes. In the future, more studies need to be conducted, including with a larger sample size, to assess the role of different variables, including patients' characteristics and medication records, on prognosis.

## Conclusions

In conclusion, this meta-analysis provides valuable insights into the mortality outcomes of patients with reduced ejection fraction (EF) compared to those with preserved EF undergoing coronary artery bypass grafting (CABG). The pooled analysis of studies demonstrated a significantly higher rate of both early and late mortality in patients with reduced EF, emphasizing the importance of EF assessment in risk stratification for CABG patients. Understanding the impact of reduced EF on surgical outcomes is crucial for better risk stratification and treatment planning in this unique patient cohort. With ongoing advancements in medical care and further research, we can strive to improve outcomes and quality of life for patients with reduced EF undergoing CABG.
